# Abstract of the Proceedings of the Buffalo Medical Association

**Published:** 1864-01

**Authors:** J. F. Miner

**Affiliations:** Secretary


ART. IV.—*Abstract of the Proceedings of the Buffalo Medical Association.*

TUESDAY EVENING, December 1st, 1863.

*Dr. Congar,* President, in the Chair; minutes of the last meeting read and accepted.

*Dr. Lockwood* presented pathological specimen, and read the following paper:

M. J. Wright, aged 46, about fifteen months since received a slight blow on his testicle during his work as a shoemaker, and his attention was called to it by thinking it unusually tender, when upon examination it was found to be enlarged. A physician was consulted and treatment received for some three months, or more, with the view to remove the enlargement. It continued to gradually increase in size, was but slightly tender on pressure, and not much painful. A dragging sensation from the increased weight was all the inconvenience he suffered.

About this time the case came under my observation, when the *gland* was found about double the natural size, hard, elastic and roughened, or uneven upon the surface. The cord was not enlarged, but the weight of the gland and other cause, had apparently elongated it, so that it hung low in the vaginal tunic of the scrotum. Persevering efforts were made to reduce the gland to its natural size, or at least to prevent its growth, but without avail. Mercury, iodine, blisters, leeches, compression and all other suitable remedies were faithfully tried with the view to overcome the disease, including confinement to a horizontal position; plain low diet, as well as outdoor exercise, tonics and stimulants, so as to be satisfied if possible that medicine could not remove the enlargement or prevent its continued increase.

A few months since I placed the patient in a dark room, had him hold a candle, burning brightly, close to the side of the scrotum, and grasping the posterior part of the swelling, so as to render its fore part as tense as possible, then by looking at the swelling from the side opposite to the candle, and placing my left hand on the fore part of the scrotum could plainly discover at superior portion of the tumor, a transparency which I regarded as serum, and thought possibly the case might be hydrocele, rather than chronic inflammation or hypertrophy.

The case was now visited by Dr. Miner in consultation, and a trocar was introduced into the transparent portion of the tumor with perhaps the escape of two ounces of clear serum. It was however, manifest before this exploration that the tumor was not caused by the accumulation of serum alone, but that the gland had suffered organic change. Efforts to improve the general condition of the system were continued for several weeks, affording no relief, and the condition both of the gland and patient constantly growing worse, I again asked the advice and assistance of Dr. Miner, who removed by operation the entire mass, which I have presented for observation in connection with this brief history.

Upon examination the gland is found to have cystic degeneration at one extremity, and in the body of the organ will be observed the characteristic appearance of malignant disease, so far at least as such appearance can be distingnished by the naked eye. It is softened in the center, and there is no appearance of anything belonging to any of the tissues of a healthy gland. This unhealthy mass is surrounded by dense fibrous tissue, while at the other extremity are two cysts; one contained perhaps an ounce of yellow serum, and the other about the same quantity of a fluid resembling ink. This pigmentary fluid may still be observed adhering to the blackened walls of the cyst which contained it. That it is malignant in character the practiced observer would almost unhesitatingly affirm by the general appearance independent of any microscopical examination; indeed if it should be found to lack the unmistakable microscopical evidence of malignancy, still very little doubt would be entertained as to its true character. Fortunately however for the diagnosis a microscopical examination made by Dr. Lothrop fully confirms, and shows, the almost unequivocal evidences of malignant degeneration.

*Dr. Lockwood* also remarked that though he had failed in his treatment in this case, still he thought that malignant disease, in its early stage, could be controlled by proper medication: that there was an incipient stage not usually noticed, when medicine was capable of removing the peculiar pathological condition upon which malignant disease depends, and that its later progress may be more or less controlled by proper medication; while even in the last stages it affords as much hope as the operations of surgery. Surgeons were so much accustomed to see immediate and positive results from their operations, that they naturally lose all faith and confidence in other and more gradual processes, and think medicine is useless, mainly because they have not patience to wait its slower action. Spoke of the imposbility of making early positive diagnosis, and the injurious effect of removing small undetermined morbid growths as cancerous, thus making impression upon the mind of the patient, equally injurious to his health and happiness, as disease of this nature would be, if actually present.

In suspected cases of malignant disease of the testicle, uterus, and ovary, he recommended and depended largely upon iodine and its preparations, both locally and internally. In commencing the treatment, he usually, for a time, gave alterative doses of mercury, with rest and low diet; afterwards persevered for a long time in the iodine treatment. Cases of disease of the testicle, neck of uterus, and of the ovary, were related, showing the benefit of this plan of treatment, and illustrating his views of the nature of malignant disease. He referred to Drs. Ashwell, Montgomery, and Gooch, as holding views similar to the ones he entertained.

*Dr. Miner* presented the neck of a uterus he had that day removed for cauliflower excresence. The excresence was not large since it had recently been removed by ligature by a physician in New York City. The case had been from the first under the medical care of one of the most experienced and capable physicians of the city, and every proper means used to overcome the disease or stay its progress. It was presented to the Society for the purpose of showing the appearance of well marked disease of this nature, and also to show how, to all appearance, the disease has been completely removed, the amputation being wholly above it, and in perfectly healthy tissue.

Under the microscope are seen large, irregular, pale cells, multiform in shape with several nuclei, such as characterize epithelial growths. Of the character of this disease, there has never been any doubt; it has not in the least disturbed the general health and has appeared to be wholly a local affection.

To remove the excrescence simply, either by caustics or ligature, would in his estimation promise nothing; possibly the milder caustics in the early stages might retard the growth, but beyond this could be productive of no good; while the ligature of the excrescence when it had become large and filled the upper portion of vagina, leaving the surface from which it grew untouched, was dangerous, unscientific and unjustifiable.

How much may be expected from the amputation of the neck of the uterus wholly above the disease, he was unprepared to state, and if he was to give an opinion it would be a guarded one, though epithelial cancer removed from many locations did not often-times return—was the least liable to return of any form of maglignant disease.*

Dr. Lockwood had claimed for the influence of medicine a great deal more than he thought it deserved; indeed was willing to confess that he did not regard medicine of the least value in either curing malignant disease, in its early stages, or arresting its progress. Was aware that arsenic, mercury and iodine had been regarded as useful in some forms of malignant disease, but thought their failure had been so uniform, and complete, as to leave no just grounds of confidence in their value. Though he would agree with him in the uncertainty of permanent relief from surgical interferance, he would yet unhesitatingly express the opinion, that cancer, though often interrupted in its growth, and progress, by natural and unknown causes, and hastened and developed by influences not understood, was still present in the system as cancer, and beyond the curative influence of medicine.

*Dr. Strong* remarked that this was a subject upon which much might be said upon both sides. It was an important question whether malignant disease could ever become benign. Had observed as Dr. Lockwood had, that Iodine was almost specific for the cure of enlarged and diseased testicle, though he could not say, that the cases cured by it, were actually malignant. Had often succeeded in removing conditions of these glands which appeared like, and were regarded by many as probably malignant. Thought to argue that malignant disease was malignant always, might be pernicious, since it would dissuade all effort and thus consign to operation some cases which would recover under proper medication. It was well to persevere with remedies until all doubt is removed, or until medication had received fair trial.

Differed somewhat with Dr. Miner, and thought it an unsettled and important question, whether this or any other form of malignant disease cannot at some stage be cured by medicine. The opinions of surgeons should be

* The surface has healed without accident of any kind, and the patient is now, (seven weeks after operation,) to all appearance, perfectly free from disease.

taken with many grains of allowance, concerning the efficiency of medicines; they rely too much upon operative means, to judge fairly of medicine. Thought that disease similar to that presented by Dr. Lockwood should not be early consigned to the knife.

*Dr. Cronyn* thought it generally admitted that malignant tumor was not always originally malignant. All forms of benign tumor may, under certain circumstances, become malignant; this he supposed established.—Injury would set up inflammatory action, giving rise to a cyst which will eventuate in malignant disease. It is strictly local for a time, and medicine should now be tried. Injury of the breast gives rise to hardness, and the cautious surgeon removes it, and thus prevents the tumor from becoming malignant.

Mentioned a case where ulceration had taken place, and he had advised to let it alone, but was surprised to learn that a distinguished surgeon had removed it; the patient died soon after. Spoke, also, of the hereditary character of cancer and related illustrative cases.

*Dr. Miner* did not regard it settled that benign tumors degenerate into malignant ones, or that injury, blows, falls, &c., were often or ever the cause of cancerous growths. Thought it much more probable that tumors which became clearly cancerous, were malignant from the beginning, and that the causes of cancer were quite separate and independent of blows or injuries.

*Dr. Peters* inquired if Dr. Cronyn thought a blow would produce cancer in a previously healthy person?

*Dr. Cronyn* was prepared to say, that injury has produced cancers in cases where there had been no previous suspicion of disease.

*Dr. Strong* thought blows often kindled inflammations which degenerate into malignancy. Did not know how it could be doubted that injury to the breast or testicle would produce inflammation, which would often degenerate into malignant disease.

*Dr. Rochester* spoke of the difficulty and imposibility of distinguishing malignant disease of testicle from scrofulous degeneration, by microscopic or other examination. Did not know but the one presented by Dr. Lockwood was cancerous, but did not think microscopic examination sufficient to decide its true malignancy. Had seen testicles affected with scrofulous degeneration removed by operation, and thought it a safer and better method than that of ulceration, which was sure to follow, and was slow, and uncertain in its termination, and liable to occasion protracted suffering. As to the character of the disease in the specimen presented by Dr. Miner, thought there could be no doubt.

*Dr. Rochester* reported the prevalence of influenza with suffocative catarrh. Had been called in the night by patients alarmed on account of great difficulty of breathing, or a feeling of suffocation. The attack on children had been, in some instances, preceded by convulsion, for which no other causes could be discovered; there was no eruptive disease to account for convulsions. Drs. Lockwood, Samo and Strong reported the same, and also noticed some other diseases as more common than usual.

*Dr. Strong* had seen several cases of the suffocative catarrh which had also ulcerative aphtha—that disease which irregular practitioners so commonly call diphtheria.

J. F. MINER, *Secretary.*

